# Arousal vs. Relaxation: A Comparison of the Neurophysiological and Cognitive Correlates of Vajrayana and Theravada Meditative Practices

**DOI:** 10.1371/journal.pone.0102990

**Published:** 2014-07-22

**Authors:** Ido Amihai, Maria Kozhevnikov

**Affiliations:** 1 National University of Singapore, Psychology Department, Singapore, Singapore; 2 Martinos Center for Biomedical Imaging, MGH & Harvard Medical School, Charlestown, Massachusetts, United States of America; Carnegie Mellon University, United States of America

## Abstract

Based on evidence of parasympathetic activation, early studies defined meditation as a relaxation response. Later research attempted to categorize meditation as either involving focused or distributed attentional systems. Neither of these hypotheses received strong empirical support, and most of the studies investigated Theravada style meditative practices. In this study, we compared neurophysiological (EEG, EKG) and cognitive correlates of meditative practices that are thought to utilize either focused or distributed attention, from both Theravada and Vajrayana traditions. The results of Study 1 show that both focused (Shamatha) and distributed (Vipassana) attention meditations of the Theravada tradition produced enhanced parasympathetic activation indicative of a relaxation response. In contrast, both focused (Deity) and distributed (Rig-pa) meditations of the Vajrayana tradition produced sympathetic activation, indicative of arousal. Additionally, the results of Study 2 demonstrated an immediate dramatic increase in performance on cognitive tasks following only Vajrayana styles of meditation, indicating enhanced phasic alertness due to arousal. Furthermore, our EEG results showed qualitatively different patterns of activation between Theravada and Vajrayana meditations, albeit highly similar activity between meditations within the same tradition. In conclusion, consistent with Tibetan scriptures that described Shamatha and Vipassana techniques as those that calm and relax the mind, and Vajrayana techniques as those that require ‘an awake quality’ of the mind, we show that Theravada and Vajrayana meditations are based on different neurophysiological mechanisms, which give rise to either a relaxation or arousal response. Hence, it may be more appropriate to categorize meditations in terms of relaxation vs. arousal, whereas classification methods that rely on the focused vs. distributed attention dichotomy may need to be reexamined.

## Introduction

In spite of the increasing interest in meditation, evidenced by its global popularity and output of scientific papers, the failure to accurately define or categorize different types of meditations has been consistently mentioned in the scientific literature [1]. One of the reasons for these difficulties stems from an insufficient understanding of the theoretical and cultural differences between different meditative traditions, which has led to inconsistent findings in the scientific literature about the nature of meditation and its neurophysiological correlates [1]. The first major attempt to provide an operational definition of meditation was proposed by Benson [2] who reported that meditation activated the parasympathetic nervous system, and described the effect of meditation as a “relaxation response”. The relaxation response refers to a physical state of deep rest, physiologically defined as a decrease in sympathetic activity (decreased heart and respiratory rate, blood pressure, oxygen consumption and reduction in cortisol and noradrenaline). Based on Benson's approach, an evolutionary theory was proposed, where meditation was viewed as a wakeful metabolic state of parasympathetic dominance – a state of deep bodily rest – similar to hibernation, where the potential of acute mental ability nevertheless remains [3].

Furthermore, a number of early studies, consistent with Benson's approach, showed that other relaxation techniques, such as self-hypnosis and progressive relaxation, produce the same reduction effect as meditation on heart and respiration rates, as well as systolic and diastolic blood pressure [4]–[8]. However, most previous studies on the physiological, electrophysiological, and neural correlates of meditation, including research that demonstrated a relaxation response, have been conducted on *Theravada* or *Mahayana* Buddhist styles of meditation. On the other hand, studies on meditative practices of the *Vajrayana* tradition (also referred to as *Tantric Buddhism*), which is central to Tibetan Buddhism (see Supporting Information S1), have been relatively limited in scope. Indeed, previous research focused primarily on Theravada meditation styles such as Shamatha or Vipassana [6], [9], both of which emphasize avoiding discursive thought by letting the practitioner concentrate on the object of meditation or his/her own mental activity, respectively [10]. In addition to Shamatha and Vipassana, which are the main meditative techniques of Theravada Buddhist schools, “compassion meditation” received much attention in recent scientific studies [11]–[13]. This type of meditation, however, pertains to all the Buddhist traditions, and it is not unique to Theravada or Vajrayana [14], [15].

While meditative techniques of all Buddhist teachings stress liberation from all conceptual delusions, the means of achieving it are quite different. Specifically, Buddhist texts emphasize that Theravada styles of meditation, such as Shamatha or Vipassana, are techniques that emphasize “internally steadying” or stabilizing the “unstable mind”, and cultivating the state of quiescence and tranquility, through which the nature of the mind could be seen without obstruction [16], [17]. Vajrayana, in contrast, emphasizes the training “which is not exactly the same as keeping the mind still and quiet” [18], but rather aims at the realization of ‘self-existing wakefulness’ or ‘an awake quality’ of the mind, free from dualistic thoughts, which is “like a radiant flame of a candle which exists all by itself” [19]. Furthermore, Vajrayana teaching emphasizes that the preoccupation with “being too calm” blocks “the recognition of self-existing wakefulness”, and that in a Vajrayana context, “it is sometimes said that stillness is not absolutely necessary…” [20]. Thus, from a Vajrayana perspective, the conceptualization of meditation as a relaxation response seems to be incongruent with Tibetan views of Vajrayana Tantric practices, which do not presuppose relaxation [21]. Indeed, Vajrayana “generation stage” practices, such as “visualization of self-generation-as-Deity”, which are to precede the “completion stage” practices pertaining to realization of emptiness (Rig-pa) are aimed at achieving a wakeful state of enhanced cognition and emotions through the use of visual imagery and the emotional arousal associated with it, when the practitioner is required to imagine his/her mind, emotions, and feelings as the ones of a specific Deity [14], [22].

Indeed, Benson [23] himself reported a contradictory and “unclear” phenomenon, that two of the three g-tummo practitioners from the Vajrayana tradition who participated in his research exhibited an activation of the sympathetic system as evidenced by increased metabolism and oxygen consumption, which is consistent with arousal but not with a relaxation response. Similarly, Corby and colleagues [24] showed that Hindu Tantric practices, which share some commonalities with Vajrayana Tantric practices, generated neural activity that promotes increased Alpha power in Electroencephalographic (EEG) recordings, as well as a small heart rate increase during meditation, suggesting the possibility of arousal rather than a relaxation response.

Based on the above review, we suggest that Vajrayana meditative practices could be described more accurately as generating arousal rather than a relaxation response. In contrast to relaxation, arousal is a physiological and psychological state of being awake or reactive to stimuli. It is characterized by an increase in the activity of the sympathetic system, which is followed by the release of epinephrine and norepinephrine from the endocrine system [25]–[27], and results in the state of *phasic alertness*, a significant temporary boost in the capacity to respond to stimuli [28]–[30]. This is in contrast to *tonic alertness*, which indicates a state of optimal vigilance where attention is sustained for a prolonged period of time. While tonic alertness can happen concurrently with relaxation, and indeed has been reported to occur during Theravada styles of meditation [31], phasic alertness is a result of the activity of the sympathetic system, and is elicited by different neurophysiological and cognitive mechanisms than tonic alertness, which are inconsistent with the state of relaxation. Thus, the first goal of this research was to examine whether Vajrayana meditative practices indeed lead to arousal, as reflected by sympathetic activation and behavioral markers of phasic alertness, such as an immediate boost in performance on cognitive tasks, instead of a relaxation response that would characterize meditative practices of the Theravada tradition.

One of the more recent approaches to characterize meditation has been to define a type of meditation in terms of the attentional mechanisms that are engaged during its practice. Several researchers [11], [32] have proposed two broad categories of meditative practices. The first category, termed Focused Attention (FA) meditation, includes meditative practices that require prolonged focused attention on a certain object, process, or state of mind. Specifically, Shamatha (Theravada style), where a special emphasis is given to an object of meditation and Deity-yoga practice (Vajrayana style) which involves holding the focus of attention on an internally generated image of a Deity, have been classified by a number of researchers as FA type of meditation [9], [33]. The second category, Open Monitoring (OM) meditation techniques, do not require sustained attention on a particular object, process, or mental state, but rather that the practitioner would view his or her cognitive states from a disengaged point of view, developing a detached awareness of his or her feelings and thoughts [11]. Examples of OM include Vipassana meditation (Theravada), which was classified by previous researchers as belonging to this category because, as stated in [34], it emphasizes “open, nonjudgmental awareness of the sensory and cognitive fields and include a meta-awareness or observation of the ongoing contents of thought” (see also [9], [11], [35]), as well as Open Presence (Vajrayana style) meditation, in which the meditator is instructed to evenly distribute attention and to not direct his or her attention toward any particular object or experience [33].

In spite of the extensive use of the above classification, the empirical support for the focused attention vs. distributed attention categorization method has been inconclusive. In one study, Manna and colleagues [36] recorded fMRI signals during Shamatha (FA), Vipassana (OM), and during rest. They reported enhanced activity in the frontal cortex and reduced activity in the prefrontal cortex in Shamatha meditation relative to rest, and enhanced activity in the prefrontal cortex during Vipassana meditation relative to rest. In contrast, another fMRI study [13] reported that FA (Shamatha) was associated with enhanced and not reduced activity in the prefrontal cortex. Moreover, in an EEG study conducted by Dunn et al. [32], the authors reported decreased Theta power over the entire scalp during both Shamatha and Vipassana relative to rest, while Cahn, Delorme and Polich [37] observed a frontal Theta increase during Vipassana. Hence, there is no unequivocal evidence that supports the fact that FA and OM differ in terms of their neurophysiological substrates. The inconsistencies in the empirical studies may be attributed to that fact that not only from a Buddhist perspective, but also from a scientific standpoint, the classification of meditations into FA and OM seems to be an oversimplification of the different processes that are involved in meditative practices. Many meditation practices are complex, and require both focused and distributed attention. For instance, Deity-yoga meditation, although classified as FA due to the emphasis on focusing on a single object of meditation [33], also requires that during the meditative practice, the meditator would be continuously mindful of the symbolic meaning of the Deity's entourage, the ornaments and environment around Deity, as well as the cognitive, physical, and emotional states of the Deity [38]. Similarly, during Vipassana meditation, the practitioner does attend to individual objects and states of mind arising in his/her awareness using focused attention. Thus, the second goal of this study was to reexamine the validity of the FA-OM classification that distinguishes between different types of meditation based on the attentional systems that they involve, versus the classification that distinguishes between meditative practices from different traditions (Theravada or Vajrayana) resulting in arousal vs. relaxation responses.

In the present paper, four different types of meditative practices were compared: two types of Vajrayna practices: Visualization of self-generation-as-Deity (FA) and Rig-pa (OM) and two types of Theravada practices: Shamatha (FA) and Vipassana (OM). We collected Electrocardiographic (EKG) and Electroencephalographic (EEG) responses (Study 1) and measured behavioral performance on cognitive tasks (Study 2) using a participant pool of experienced Theravada practitioners from Thailand (Yannawa Temple, Bangkok) and Nepal (the International Buddhist Meditation Center and Amarapura Buddhist Nunnery, New Baneshwor) and Vajrayana practitioners from Nepal (Shechen monastery, Kathmandu). In order to measure the physiological correlates of arousal and relaxation responses, in Study 1 we used EKG measures, which have been shown to be reliably related to the activity of autonomic system [25], [39]–[42]. Since relaxation type responses are expected to increase parasympathetic activity, meditations that produce relaxation should elicit the following EKG markers indicative of parasympathetic activation: 1) an increase in high-frequency power (HF), 2) a lower ratio between low and high frequencies (LF/HF) [25]. We did not use other conventional physiological measures of the output of the autonomic system, such as skin conductance resistance (GSR) or respiration rates, as the validity of these measures could not have been established in the present research. Respiration rates are highly influenced by certain types of meditations, which intentionally utilize breathing in order to alter the levels of relaxation and arousal, and could therefore present a mediating factor to EKG changes rather than an independent measure of the state of arousal or relaxation. GSR is highly influenced by humidity and temperature and could not be reliably measured in the present experimental conditions [43], [44]. To complement EKG measures of autonomic system activity used in Study 1, in Study 2 we measured changes in performance on cognitive tasks following Vajrayana and Theravada styles of meditation as a behavioral marker of phasic alertness, which occurs during increased arousal. We expected to find an immediate significant increase in cognitive performance following Vajrayana but not Theravada styles of meditation.

Furthermore, to examine the validity of the FA-OM classification that distinguishes between different types of meditations based on the attentional systems they involve, versus the classification that distinguishes between meditative traditions (Vajrayana-Theravada), we compared EEG data recorded during each type of meditation. We expected that the neurophysiological correlates of the four types of meditations examined in this study would differ significantly across traditions but not across the FA-OM classification.

## Methods

### Study 1

Since Vajrayana and Theravada practitioners are not trained in the same meditative practices, a within-subjects design was not feasible. As a consequence, we used a mixed design, with the data from the Theravada style practitioners recorded at Yannawa Temple (Bangkok, Thailand), and the data from the Tibetan Vajrayana style practitioners recorded at the Shechen Monastery (Kathmandu, Nepal).

#### Participants

Ten long-term Theravada style practitioners who practice meditation at Yannawa Temple in Thailand, Bangkok (mean age  = 41.4, 2 females), with an average of 8 years of meditation experience participated in the study. In addition, nine long-term Vajrayana practitioners (mean age  = 47.5, 1 female), with an average of 7.4 years of meditation experience from Shechen monastery in Nepal participated in the study. All of the participants reported having no cardiovascular conditions, and were free of medication for the duration of the study. The subjects provided written, informed, consent for their participation in the study. The study was approved by the National University of Singapore's review board, which implements commonly used procedures for data deposition.

#### Theravada practices

In Buddhist scriptures, Shamatha practice relates to training in the concentration of attention. During the practice of Shamatha, the practitioners are instructed to place undistracted attention on the object of meditation, while withdrawing their focus from other objects [45]. Vipassana refers to insight into the true nature of reality, entailing an understanding of the impermanence of everything that exists, which is coupled with pacification (serenity) of the mind [17]. In meditation research, Vipassana practice typically begins with Shamatha (with a focus on the breath), but when awareness wanders away from the meditator's breathing, he or she is instructed to recognize that the mind has wandered, as well as the content that is currently occupying his or her mind [9].

**Figure 1:**
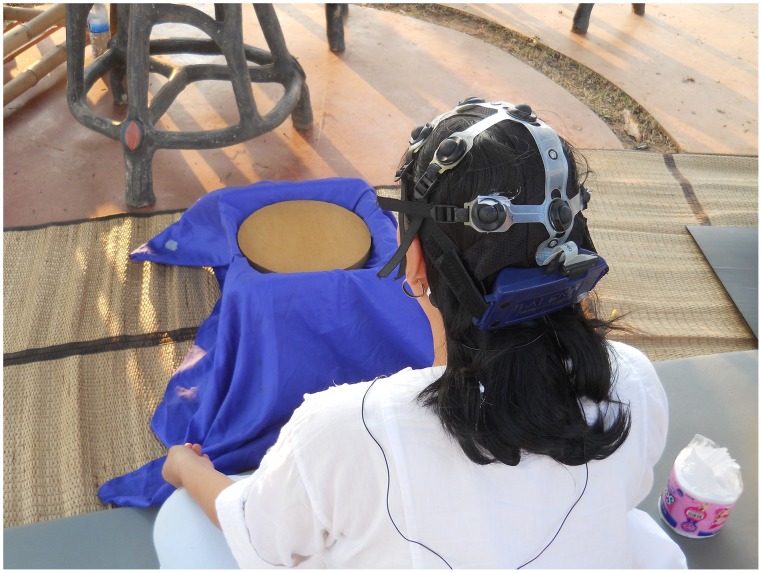
Participant meditating on earth Kasina while her EEG and EKG recordings are taken.

For the purpose of this research, we used Kasina meditation as a particular type of Shamatha practice. Kasina meditation refers to objects of meditation that possess certain characteristics described in the Pali Tipitaka [46]. Kasina objects of meditation are typically colored disks, which differ from each other in terms of their color, size, object composition and other properties, depending on the type of Kasina used. The Pali Tipitaka [46] describes the following most commonly used Kasinas: earth, water, fire, air, blue, yellow, red, and white (see **Figure 1**). In the current study, Kasina was used in place of the more popular Shamatha practice where a meditator focuses on the breath for a long period of time [9] to dissociate it more easily from Vipassana, which implements focusing on the breath.

#### Vajrayana practices

**Figure 2:**
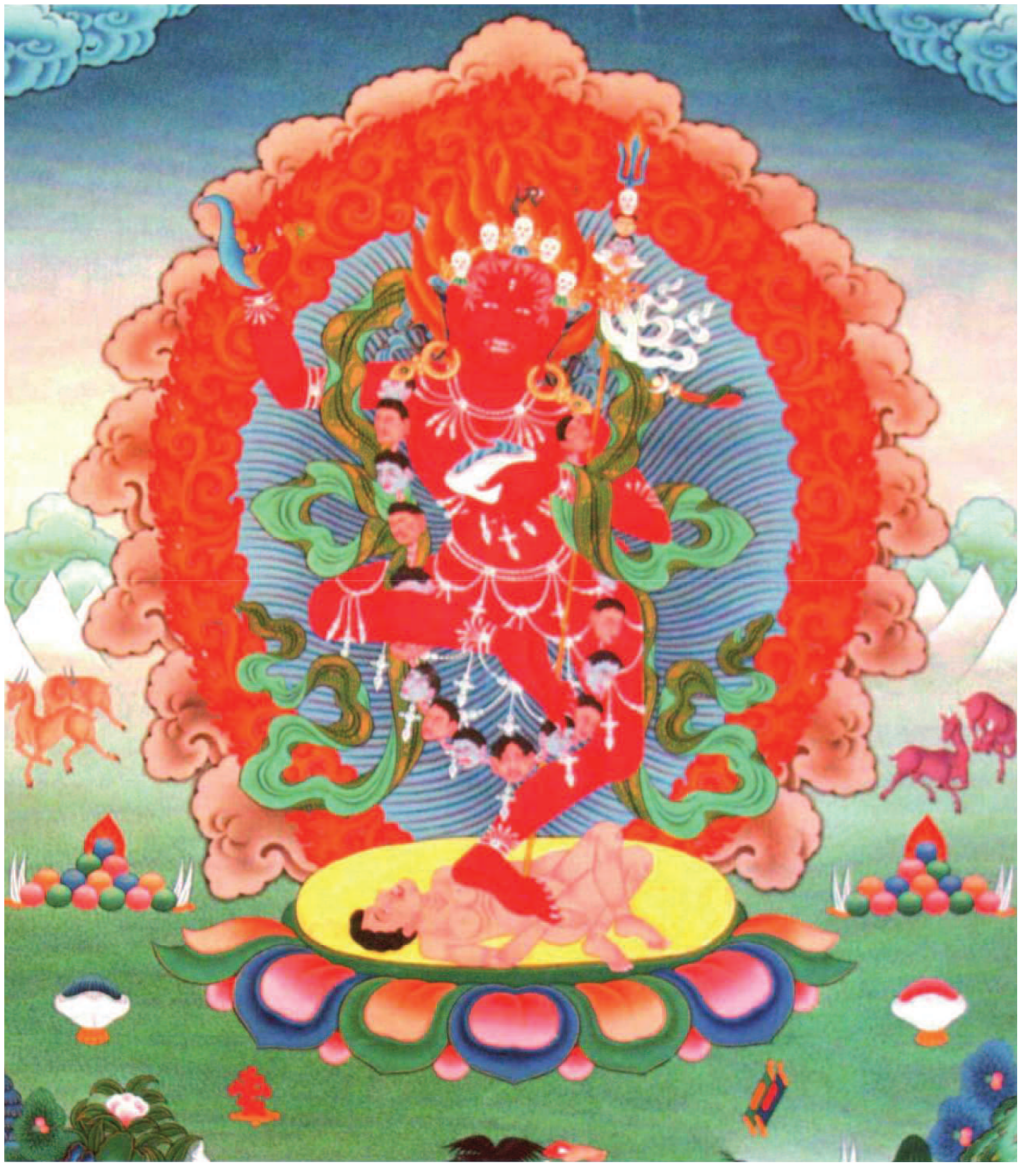
An example of a meditation Deity (Vajrayyogini) used by the participants in the practice of Deity Visualization.

Visualization of self-generation-as-Deity practice (Tibetan “Kyerim”; hereafter referred to as Deity meditation) originated in Hindu and Buddhist Tantric traditions in India and was later adopted by Tibetan Buddhism [47]. The practice involves holding the focus of attention on an internally generated image of a Deity surrounded by his or her entourage (see **Figure 2**). The content of Deity meditation is rich and multimodal, requiring the generation of colorful three-dimensional images (e.g., the Deity's body, ornaments, and environment), as well as representations of sensorimotor body schema, feelings, and emotions of the Deity. The image temporarily replaces one's sense of self and internal perception of the real world [38]. In Vajrayana, visualization of oneself as a Deity is related to the *generation or development stage*, which is the first stage of the meditation practice [48].

During Rig-pa meditation, which follows the final stages of Deity meditation, and represents the *completion stages* of the meditative practice [14], a meditator visualizes the dissolution of the Deity and its entourage into emptiness, and aspires to achieve awareness devoid of conceptualization. While performing Rig-pa, the meditator attempts to evenly distribute his or her attention so that it is not directed toward any object or experience. Although various aspects of experience may arise (e.g. thoughts, feelings, images, etc.), the meditator is instructed to let them subside on their own, without dwelling on them or examining them [49], [50]. The important distinction between Vipassana and Rig-pa is that Rig-pa is considered to be a meditative practice with no object of meditation; it does not require noticing or watching the content of attention, the activity that is associated with a dualistic mind, but only to be fully aware of it [14].

#### Procedure

The data from the Theravada style practitioners were recorded in a meditation hall at the Yannawa Temple (Bangkok, Thailand), and the data from the Tibetan Vajrayana style practitioners was recorded at the Shechen Monastery library (Kathmandu, Nepal). EEG and EKG were continuously recorded throughout the study. At the beginning of the session, each participant performed a 10 minute Rest condition, during which they were explicitly instructed not to meditate but to remain seated with their eyes closed, and to simply relax. Following a 5 minute break, the Theravada practitioners were asked to perform 15 minutes of Shamatha meditation followed by 15 minutes of Vipassana. Tibetan Vajrayana practitioners were asked to perform 15 minutes of Deity meditation after the Rest condition, followed by 15 minutes of Rig-pa meditation. The orders of meditation were chosen per request of the participants who found that it is more natural first to meditate in Shamatha followed by Vipassana. Similarly, since Rig-pa (completion stages) follow the end of Deity practice (generation stage), Vajrayana practitioners performed Deity first followed by Rig-pa. For participants who did not speak English, interpreters translated all the instructions into their native language.

In contrast to Theravada styles of meditation, which are performed with closed eyes, Vajrayana practices are often performed with open eyes. However, to make the experimental conditions as similar as possible, we instructed all the practitioners to meditate with closed eyes. Importantly, we ensured that all our Vajrayana practitioners were comfortable with such a request, and they all confirmed that this would not affect their meditation.

#### EEG and EKG Recordings and Protocol

EEG was continuously recorded at the POz,Pz,Fz,C3,C4,F3,F4,P3 and P4 scalp regions, positioned according to the standard 10/20 system [51] using a B-Alert portable EEG cap (Advanced Brain Monitoring, Inc.), as well as from 2 additional electrodes placed on the right and left mastoids. EKG was recorded via two electrodes placed over on the right collar bone and below the left rib cage. EEG and EKG were sampled at 256 Hz, and referenced to the average between the two mastoid electrodes. Signals showing ocular and muscular artifacts were manually excluded from the study, and a high-band pass filter of 0.1 Hz was applied to the EEG data. Moreover, a digital notch filter was applied to the data at 50 Hz to remove artifacts caused by nearby electrical devices.

#### Heart rate variability analysis

Prior to the mid-1980s, heart rate variability (HRV) was typically analyzed through time domain methods but not frequency domain methods [25], [41], [42]. It has since been established that the reliability of time domain methods is highly dependent on the length of the EKG recordings, and that they are ideal for analyzing recordings that are typically longer than 18 hours [25]. On the other hand, during short term recordings, such as the ones conducted during meditation studies, frequency domain methods can be used reliably [25]. Specifically, the frequencies used in the analysis of autonomic system activity are EKG high frequencies (HF), and the ratio of low to high frequencies (LH/HF) [25]. While some researchers [52]–[55] proposed that LF is a marker of sympathetic modulation, others attribute its activity to both sympathetic and parasympathetic influences [56], [57]. In contrast, increases in HF could be reliably attributed to the activity of the parasympathetic system [41], . Under normal circumstances, HF decreases indicate decreased parasympathetic and increased sympathetic activation [58], [59], although in some extreme cases (e.g. stress or physical exercise), increases in HF could accompany an increase in sympathetic response [58]–[60].

A cubic spline interpolation with a 500 Hz sampling rate was performed on the EKG data, in order to improve the accuracy of the heart rate variability estimations [61], [62]. Subsequently, HF and LF/HF were computed using Welch's periodogram method (FFT spectrum), and were measured in absolute power (milliseconds squared). Since a 2 minute recording period is needed to accurately assess LF/HF [25], we analyzed EKG from an additional minute preceding the 3 minute period that was used for EEG analysis. Hence, EKG sampled at 256 Hz was extracted from a 4 minute period, and interpolated to produce a 2 minute period sampled at 500 Hz. The HF frequencies were 0.15–0.4 Hz and LF frequencies were 0.04–0.15 Hz, which are the frequency ranges that are most commonly used in EKG analysis [58], [63]–[66]. HF and LF/HF were then analyzed as dependent variables in separate repeated measures ANOVAs. Since the Theravada and Vajrayana meditations were performed by separate groups of subjects, we first compared each of them to the control Rest condition using within subject ANOVAs, with Condition (Meditation, Rest) as an independent factor. Subsequently, significant effects were contrasted using a mixed design ANOVA (2×2), with Tradition (Theravada-Vajrayana) as the between-subject factor, and Attention (FA-OM) as the within-subject factor.

#### Spectral Analysis

For each electrode and 1 second epoch, the power spectral distribution (PSD) was calculated using Welch's method [67], where power values are averaged and a 512 millisecond time window is applied. Subsequently, the mean power at the Delta (1–4 Hz), Theta (4.5–7.5 Hz), Alpha (8.5–12.5 Hz), Beta (13–25 Hz), and Gamma (35–44.5 Hz, 60–95.5 Hz, 110–128 Hz) frequencies were used as the dependent variables in the analyses. Importantly, we analyzed only a 3 minute epoch at the end of the meditation period, during which the meditators were most likely to be in a deep meditative state.

Theravada and Vajrayana meditations were first analyzed using within subject ANOVAs, for which the independent factors were Condition (Meditation, Rest) and Location. In order to test for potential effects of hemisphere (laterality), which are often observed [68], we divided the scalp into 3 regions, each of which consisted of the average of 3 electrodes that were selected according to their location: Left – C3,F3,P3; Right – C4,F4,P4; Center – Cz,Fz,POz. Subsequently, significant effects were contrasted using a mixed design ANOVA (2×2×3). The between-groups factor was Tradition (Theravada-Vajrayana), and the within-subject factors were Attention (FA-OM) and Location (Left, Right or Center).

#### Coherence Analysis

The mean squared coherence was measured between electrodes F3 and F4 (Frontal), the average between F3 and F4 and the average between electrodes C3 and C4 (Fronto-Central), P3 and P4 (Posterior), and the average between P3 and P4 and the average between F3 and F4 (Fronto-Posterior), separately for Alpha, Beta, and Gamma power. Subsequently, each one of the 12 frequency-coherence combinations (Alpha: Frontal, Fronto-Central, Posterior, Fronto-Posterior; Beta: Frontal, Fronto-Central, Posterior, Fronto-Posterior; Gamma: Frontal, Fronto-Central, Posterior, Fronto-Posterior) was separately analyzed as the dependent variable in a repeated measures ANOVA, with Condition (meditation vs. rest) as the independent factor. Furthermore, significant results were analyzed in a mixed ANOVA (2×2) using Attention (FA vs OM) as the within-subject factor, and Tradition (Vajrayana vs Theravada) as the between groups factor.

### Study 2

In Study 2 we investigated whether the practice of Vajrayana or Theravada types of meditation will elicit an immediate significant enhancement in cognitive tasks as a result of an increase in phasic alertness, associated with arousal [28]–[30]. Indeed, previous research demonstrated a positive correlation between arousal and cognitive performance [69]–[71], so that the amount of pre-stimulus arousal predicted the probability of success on a number of visual tasks, including attention and memory guided delay tasks. Furthermore, a dramatic temporary enhancement (of about 15–20 minutes duration) on visual tasks such as dynamic spatial transformations and the maintanence of a static image in visual working memory, was previously demonstrated to occur in experienced Vajrayana practitioners immediately following 20 minutes of Deity meditation practice [33].

Similarly to [33], we used a between-subject design, in which meditators were administered two computerized tasks assessing different aspects of visual processing (mental rotation and visual memory tasks) before and immediately after a 20 minute meditation session. Since we were interested in the effect of each type of meditation on cognitive performance, in contrast to Study 1, where EEG and EKG measures were taken from Vajrayana practitioners who performed Rig-pa meditation immediately after Deity, and from Theravada practitioners who performed Vipassana right after Kasina, in Study 2 each participant performed only one type of meditation practice (Kasina, Vipassana, or Rig-pa. As explained below, data from Deity practitioners was taken from a previously published study [33]). We hypothesized that Vajrayana types of meditation would lead to an immediate improvement in these tasks following meditation practice, due to an increase in phasic alertness associated with arousal. On the other hand, Theravada meditators should not significantly differ in their performance on these tests before and after meditation.

#### Participants

As in Study 1, all participants reported having no cardiovascular conditions, and were free of medication for the duration of Study 2. The first (Kasina) group of participants consisted of 12 long-term Theravada practitioners from Yannawa Temple, Bangkok (mean age  = 43.2, 2 female) with an average of 10 years of meditation experience (10 practitioners from this group participated in Study 1). This group of participants performed Kasina meditation. The data of this group were collected at the Yannawa Temple (Bangkok, Thailand).

The second (Vipassana) group consisted of 14 Theravada practitioners who were asked to perform Vipassana meditation (mean age  = 43.2, 4 female) with an average of 12.3 years of meditation experience. The ten male practitioners were recruited from the International Buddhist Meditation Center (Sankhamul, New Baneshwor, Nepal), and the four female practitioners were recruited from the Amarapura Buddhist Nunnery in Nepal. The data of this group of participants were collected at the International Buddhist Meditation Center (New Baneshwor, Nepal).

The third (Rig-pa) group consisted of the same 9 long-term Vajrayana practitioners from the Shechen monastery in Nepal (mean age  = 47.5, 1 female) who participated in Study 1, and have an average of 7.4 years of meditation experience. The data from this group of participants were collected at the Shechen monastery library and they were asked to perform Rig-pa meditation. Although Rig-pa is usually practiced right after Deity meditation in one session, our experimental design required that each participant would perform only one type of meditation. All participants acknowledged that they were comfortable in performing Rig-pa meditation not preceded by Deity.

The behavioral data from two visual tasks administered before and after a meditation session in the above three groups were compared with the data from the same tasks performed before and after Deity meditation, which were previously published in Kozhevnikov et al.'s study [33] that incorporated the same experimental procedure. The group of Deity meditators [33] consisted of 15 Vajrayana long-term practitioners (mean age  = 42, 5 female) with an average meditation experience of 13 years. (It should be noted that Kozhevnikov et al. [33], in addition to Deity meditators, investigated a group of practitioners who performed “Open Presence” practices, which although in some respects were similar to Rig-pa practice, included a mixture of OM meditations from different schools of Tibetan Buddhism, not necessarily Tantric).

#### Mental Rotation Test (MRT)

On each trial of the MRT, participants viewed a pair of three dimensional pictures, which were rotated relative to each other around the x, y, or z-axis. Across trials, the amount of rotation ranged from 40° to 180°, in 20° increments. Participants were required to judge whether the two pictures were of the same form, or whether the forms were mirror-reversed. The test consisted of 36 trials, 18 in which the forms were the same, and 18 in which they were mirror-reversed.

#### Visual Memory Test (VMT)

**Figure 3:**
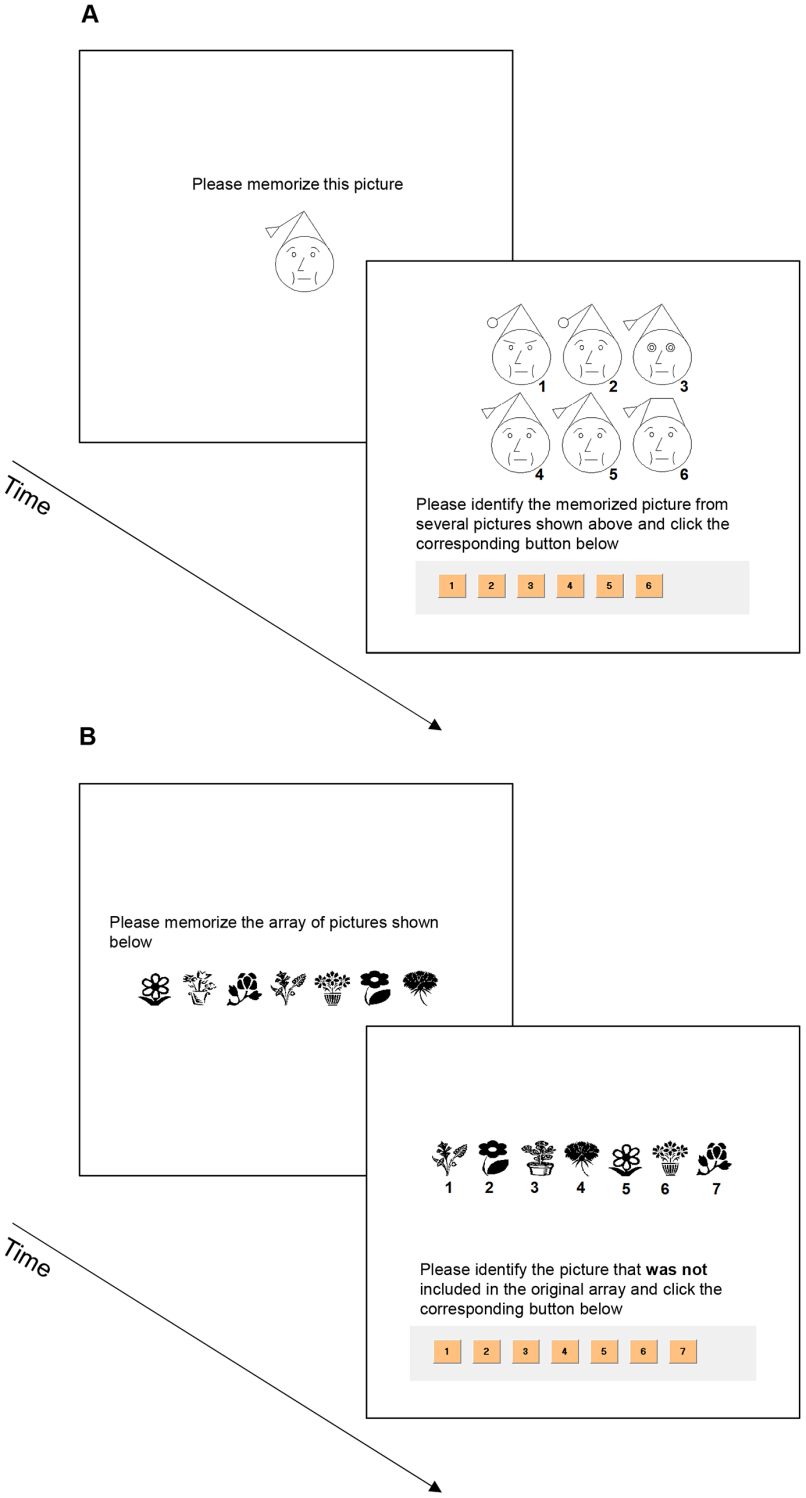
Examples of items from the Visual Memory Test.

The VMT [72] consisted of two parts. In the first part, participants performed 6 trials during which a single image first appeared for 5 seconds and was subsequently replaced by an array of six images. The array consisted of the original image along with 5 distractors, and the participants were asked to determine which image in the array was the first image (**see** **Figure 3**). The second part of the VMT consisted of 18 trials, during which participants first viewed an array of seven images that appeared for 8 seconds. This array was subsequently replaced by another array of seven images, 6 which appeared in the previous array and one novel image. Participants were asked to determine which image in the second array did not appear in the first.

#### Procedure

The procedure for Study 2 was similar to the procedure reported in [33]. All the participants were tested individually in a testing session that lasted from 1.5–2 hours. First, the participants completed the MRT and VMT pre-tests, the order of which was counterbalanced. Similarly to Study 1, for those who did not speak English, interpreters translated all the instructions into the native language of the participants before each test began. After completing the pre-test, the Theravada (Thai) participants from the Kasina group were asked to perform Kasina meditation. The Theravada (Nepalese) participants were asked to perform Vipassana, and Vajrayana practitioners were asked to perform Rig-pa. Similarly to [33], all the groups meditated for 20 minutes.

Although different groups of meditators were tested in different locations, we tried to make the testing conditions as similar as possible. In particular, during all the tests, we used a quiet room in the monasteries with moderate temperature; the same laptop computers were used during all the procedures, and the same training session and instructions (apart from the meditation instructions) were given to all the participants. Importantly, the Vipassana meditators in this study were from the International Buddhist Center in Nepal, enabling us to compare Theravada and Vajrayana (Rig-pa) meditations in a similar environment. As Kasina is not practiced in Nepal, this data was collected in Thailand.

## Results

### Study 1

#### Heart Rate Variability

**Figure 4:**
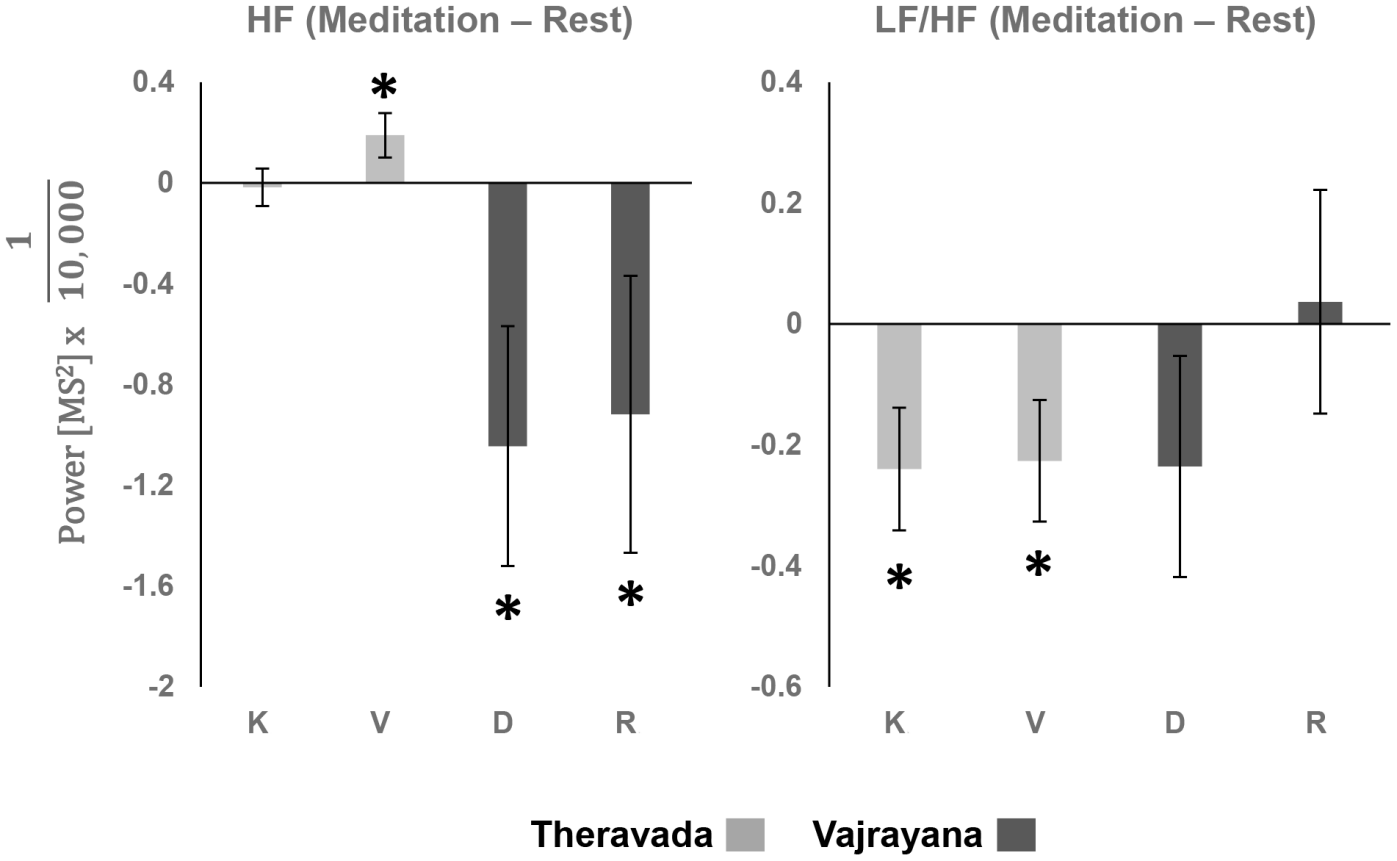
EKG differences between Meditation and Rest (K – Kasina, V – Vipassana, D – Deity, and R – Rig-pa).

The Heart Rate Variability results are summarized in Table S1. For Theravada meditation, we observed a marginally significant main effect of Condition (Rest, Kasina, Vipassana): F(2,18) = 3.2, p = 0.06. As we hypothesized that HF would increase during Theravada meditations, we performed planned pairwise comparisons between Vipassana and Rest, and Kasina and Rest. These comparisons showed that the difference between Vipassana and Rest was significant (p<0.05), whereas the difference between Kasina and Rest was not significant (p>0.4), see **Figure 4**. Furthermore, we observed a significant main effect of Condition on LF/HF, F(2,18) = 3.67, p<0.05. Since we hypothesized that LF/HF would decrease during Theravada meditations, we performed planned pairwise comparisons between Vipassana and Rest, and Kasina and Rest, which showed that the differences between Kasina and Rest (p<0.05), and Vipassana and Rest (p<0.05), were both significant. Moreover, the post-hoc comparison between Kasina and Vipassana was not significant (p>0.8). This suggests that both Kasina and Vipassana induced an increase in parasympathetic activity, corresponding to a relaxation response, with a clearer pattern of parasympathetic increase for Vipassana than Kasina, as evidenced by both an increase in HF and a decrease in LF/HF.

For Vajrayana meditation, the analysis demonstrated a marginally significant Condition (Rest, Deity, Rig-p) effect for HF, F(2,16) = 3.19, p = 0.07. As we hypothesized that HF would decrease during Vajrayana meditations, we performed planned pairwise comparisons between Deity and Rest, and Rig-pa and Rest. HF was significantly decreased in Deity relative to Rest (p<0.05) and in Rig-pa relative to Rest (p<0.05). The post-hoc comparison between Deity and Rig-pa was not significant (p>0.8), see **Figure 4**. The effect of Condition on LF/HF was also not significant, F(2,16) = 1.3, p>0.2. Overall, the HRV analysis showed that although there were no significant changes in LF/HF, there was a significant decrease in HF, which is primary marker of increase in sympathetic activity, suggesting that during both Vajrayana practices, the practitioners exhibited an arousal response.

Furthermore, the between traditions comparison showed a significant Tradition (Theravada, Vajrayana) effect on HF, F(1,17) = 7.02, p<0.05, so that HF was significantly decreased during Vajrayana in comparison to Theravada, see **Figure 4**. However, the main effect of Attention (FA, OM) on HF was not significant (F<1) as well as Attention X Tradition interaction (F<1). The between-tradition analysis of LF/HF showed non-significant main effects of Tradition: F(1,17) = 1.34, p>0.2 and Attention: F(1,17) = 2.13, p>0.1, as well as a non-significant Attention X Tradition interaction: F(1,17) = 1.77, p>0.2.

Overall, the results suggest that Theravada and Vajrayana meditative practices lead to very different patterns of HRV responses, exhibiting patterns that are consistent with a relaxation response for Theravada practices and arousal responses for Vajrayana practices.

#### Spectral analysis

The statistical results for all the frequencies are summarized in Table S2, and are described here in ascending order (Delta, Theta, Alpha, Beta, Gamma).

#### Delta

**Figure 5:**
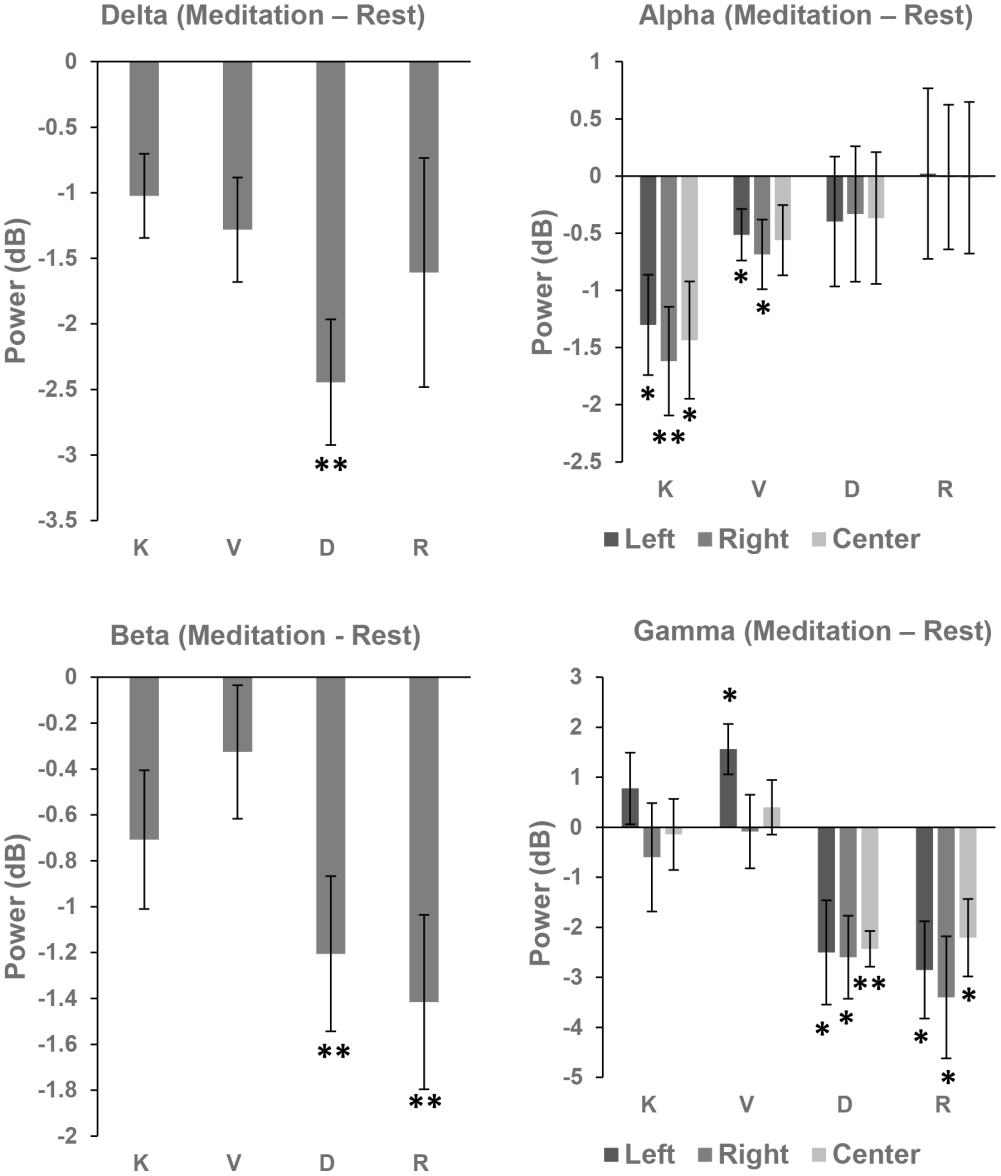
Differences in EEG frequency power between Meditation and Rest (K – Kasina, V – Vipassana, D – Deity, and R – Rig-pa).

For Theravada style of meditation, the analysis showed a significant main effect of Condition: F(2,18) = 8.37, p<0.01, so that Delta power was reduced during meditation in comparison to Rest. However, posthoc comparisons using Bonferroni adjusted α levels of 0.017 per test (α = 0.05/3 = 0.017) did not reveal significant differences between Rest, Kasina, and Vipassana. The main effect of Location was significant: F (2,18) = 7.19, p<0.01, suggesting that Delta power was greater at the Center (see **Figure 5**). The Condition X Location interaction was not significant: F(4,36) = 1.41, p>0.2.

We also found a significant main effect of Condition for Vajrayana meditation: F(2,16) = 5.11, p<0.05, indicating that Delta power was reduced during Vajrayana in comparison to Rest (see **Figure 5**). Using a Bonferroni correction (α = 0.05/3 = 0.017), we found a significant difference between Deity and Rest (p<0.001), but the difference between Rig-pa and Rest was not significant (p>0.1). There was no significant difference between Deity and Rig-pa (p>0.3). The main effect of Location was not significant: F(2,16) = 2.36, p>0.1, as well as the Condition X Location interaction: F(4,32) = 1.08, p>0.3.

The comparison between traditions showed that the main effects of Attention, F<1, and Tradition, F(1,17) = 1.97, p>0.1, were not significant, as well as the Attention X Tradition interaction, F(1,17) = 1.48, p>0.2.

#### Theta

The effect of Condition was not significant for either Theravada, F(2,18) = 1.11, p>0.3 or Vajrayana styles of meditation, F(2,16) = 2.5, p>0.1, as was the Condition X Location interaction – Theravada style: F(4,36) = 1.9, p>0.1; Vajrayana style: F<1. We found a significant main effect of Location that was unrelated to meditation – Theravada style: F(2,18) = 20.16, p<0.001; Vajrayana style: F(2,16) = 3.5, p = 0.055 – suggesting that Theta power was higher at central scalp regions for both meditation and Rest.

#### Alpha

Although meditative states were often reported to produce an increase in Alpha power [32], [73], [74], when adequate control conditions were applied to account for general relaxation, then either decreased Alpha power, or no differences between meditation and rest were demonstrated [34], [37], [75], [76]. Indeed, other neuroscience research also showed that decreased Alpha power is associated with deep relaxation, while increases in Alpha are associated with wakefulness, attention, and task load [77], [78]. Therefore, we expected to find reductions in Alpha power for Theravada meditations, and increases in Alpha power for Vajrayana practices.

For Theravada styles of meditation, the analysis revealed a significant main effect of Condition, F(2,18) = 6.84, p<0.01, indicating that Alpha power was reduced during meditation relative to Rest (see **Figure 5**). The main effect of Location was also significant: F(2,18) = 13.49, p<0.001, as was the Condition X Location interaction: F(2,18) = 6.84, p<0.01. Hence, follow up ANOVAs were performed at each Location. The effect of Condition was significant at all Locations [Left: F(2,18) = 6.49, p<0.01; Right: F(2,18) = 8.19, p<0.01; Center: F(2,18) = 5.72, p<0.05]. Moreover, the planned pairwise comparisons between Kasina and Rest were significant at all Locations [Left: p<0.05, Right: p<0.01, Center: p<0.05]. The difference between Vipassana and Rest was significant at the Left, p<0.05, and Right Location, p = 0.051, but not at the Center, p>0.1. Using a Bonferroni correction (α = 0.017), we did not find significant differences between Kasina and Vipassana at any Location.

For Vajrayana types of meditation, in contrast to our predictions, there were no significant differences in Alpha power between Deity, Rig-pa, and Rest (p>0.8 for all main effects and interactions).

The comparison between traditions showed that the main effects of Attention: F(1,17) = 2.68, p>0.1, and Tradition: F(1,17) = 1.86, p>0.1, were not significant, as well as the Attention X Tradition interaction (F<1). The main effect of Location was not significant (F<1), nor were there any significant interactions between Location and other factors (p>0.3 for all comparisons).

#### Beta

For Theravada styles of meditation, ANOVA showed a significant effect of Condition: F(2,18) = 3.68, p<0.05, demonstrating that Beta power was reduced during meditation (see **Figure 5**). The effect of Location was not significant (F<1), however the Condition X Location interaction was significant, F(4,36) = 3.77, p<0.05, suggesting that the difference between the Conditions was most prominent at the Right, followed by the Center Location (see **Figure 5**). Using a Bonferroni correction (α = 0.017), we did not find any significant difference between Kasina and Rest (p>0.03), Vipassana and Rest (p>0.15) and Vipassana and Kasina (p>0.05) at any Location.

For Vajrayana style, there was also a significant main effect of Condition: F(2,16) = 8.42, p<0.01, demonstrating that Beta power was reduced during meditation relative to Rest (see **Figure 5**). Using a Bonferroni correction (α = 0.017), we observed significant differences between Deity and Rest (p<0.01), and Rig-pa and Rest (p<0.01). However the comparison between Deity and Rig-pa was not significant (p>0.6). A marginal effect of Location was obtained: F(2,16) = 3.43, p = 0.058, suggesting that Beta power was higher over the left hemisphere for both Meditation and Rest (see **Figure 5**), and we did not find an interaction between Condition and Location: F(2,16) = 1.01, p>0.4.

The between traditions analysis demonstrated a marginally significant main effect of Tradition, F(1,17) = 3.69, p = 0.07, which suggests that Beta power was more decreased during Vajrayana than Theravada (see **Figure 5**). No other main effects or interactions were observed between traditions (p>0.1 for all comparisons).

#### Gamma

For meditations belonging to the Theravada tradition, although the main effects of Condition (F<1), and Location: F(2,18) = 1.48, p>0.2 were not significant, we found a significant Condition X Location interaction: F(4,36) = 3.09, p<0.05, and separate ANOVAs at each level of the Location factor showed a significant trend at the Left Location: F(2,18) = 2.76, p = 0.09, but not the at the Right or Center (F<1 for both comparisons). Since previous studies found increases in Gamma during Theravada meditation [37], we performed planned pairwise comparisons between both Theravada meditations and Rest at each Location. These comparisons revealed that Gamma power was increased for Vipassana relative to Rest at the Left Location (p<0.05), but not at the Right or Center (p>0.2). The planned pairwise comparisons between Kasina and Rest were not significant (p>0.3 at all Locations), as were the comparisons between Vipassana and Kasina (p>0.2 at all Locations).

As for Vajrayana, there was a significant main effect of Condition: F(2,16) = 6.16, p<0.01, demonstrating a significant reduction in Gamma power during Deity and Rig-pa relative to Rest (see **Figure 5**). Moreover, using a Bonferroni correction (α = 0.017) we observed significant differences between Deity and Rest (p<0.01), and Rig-pa and Rest (p = 0.012), but there was no difference between Deity and Rig-pa (p>0.7). We also observed a main effect of Location, F(2,16) = 6.33, p<0.01, indicative of lower Gamma power at the Center (see **Figure 5**). However this effect was not influenced by meditation, as the Condition X Location interaction was not significant (F<1).

The differences in Gamma power between Theravada and Vajrayana were significant, as demonstrated by a significant Tradition main effect: F(1,17) = 12.27, p<0.01. No other main effects or interactions were found in the between traditions analysis (p>0.1 for all comparisons).

Overall, the results show large EEG differences between Theravada and Vajrayana traditions. In particular, we observed a reduction in Alpha power for Theravada (both Kasina and Vipassana), but not Vajrayana practices. Furthermore, Gamma power was increased during Vipassana, but decreased during Vajrayana (both Deity and Rig-pa) practices. Interestingly, decreases in Beta power were observed in both Theravada and Vajrayana meditation, but they were more prominent during Vajrayana. On the other hand, we did not find any significant differences in EEG frequency power within traditions that could be explained through the FA-OM framework.

#### Coherence Analysis

**Figure 6:**
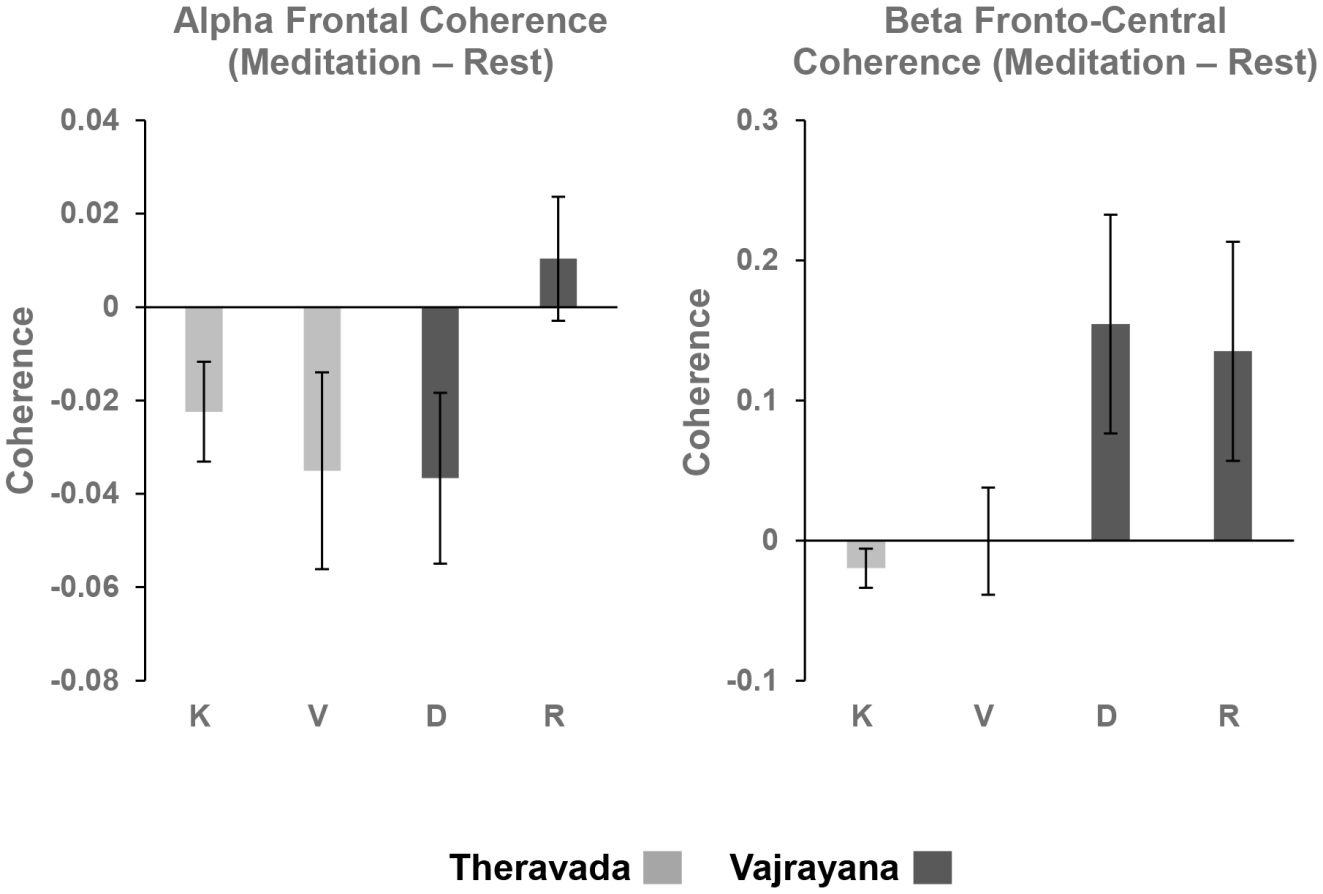
Differences in Coherence between Meditation and Rest (K – Kasina, V – Vipassana, D – Deity, and R – Rig-pa).

The statistical results are shown in Table S3. For Theravada styles, the analysis showed a marginally significant Condition effect for Alpha Frontal Coherence: F(2,18) = 2.74, p = 0.09, suggesting that Alpha Frontal Coherence may be reduced in meditation relative to Rest (see **Figure 6**). No other significant effects were found (p>0.15 for all other comparisons of Alpha, Beta, or Gamma Coherence).

For Vajrayana style of meditation, Alpha Frontal Coherence also showed a marginally significant effect of Condition, F(2,17) = 3.16, p = 0.07, suggesting that it is reduced during Vajrayana as well. In addition, for Vajrayana meditation, the analysis of Beta Fronto-Central Coherence revealed a marginally significant effect of Condition: F(2,16) = 3.34, p = 0.06, suggesting that it was increased during Vajrayana meditation relative to Rest (see **Figure 6**). No other significant effects were obtained (p>0.1 for all comparisons of Alpha, Beta, or Gamma Coherence).

The comparison between traditions showed that for Alpha Frontal Coherence, although the main effects of Attention, p>0.2, and Tradition, F<1, were not significant, a significant Attention X Tradition interaction was observed: F(1,17) = 4.98, p<0.05. This interaction shows that, whereas Alpha Frontal Coherence was greater for Rig-pa (OM) than Deity (FA) of the Vajrayana tradition, the opposite trend was observed for Theravada, so that more coherence was observed for Kasina (FA) than Vipassana (OM) (see **Figure 6**). No other effects of Alpha Frontal Coherence were observed (p>0.2 for all main effects and interactions). Moreover, the Beta Fronto-Central Coherence analysis revealed a marginally significant main effect of Tradition, F(1,17) = 4.15, p = 0.057, suggesting that it was increased during Vajrayana relative to Theravada (see **Figure 6**). No other effects of Beta Coherence were observed between traditions (F<1 for all comparisons).

In summary, the results of the coherence analysis support a tradition-based classification of meditations, but not the attention-based classification. While we found an increase in Beta Fronto-Central Coherence for Vajrayana practices relative to Rest, no such effect was found for Theravada meditative practices. Furthermore, we observed an increase of Alpha Frontal Coherence for Rig-pa (OM) relative to Deity (FA) in Vajrayana, but a decrease for Vipassana (OM) relative to Kasina (FA) during Theravada meditation, which is incompatible with the attention-based classification.

### Study 2

The results of the study are summarized in Table S4. Outlier response times (RTs±2.5 SD from a participant's mean) were deleted, which accounted to less than 3% of responses in every condition.

As in [33], to avoid issues of speed-accuracy trade-off that are often observed during visual tasks [79], we calculated the visual processing efficiency for each participant for the MRT and VMT tasks by dividing each participant's proportion of correct responses by their logarithmically transformed average reaction time (lnRT).

#### MRT

**Figure 7:**
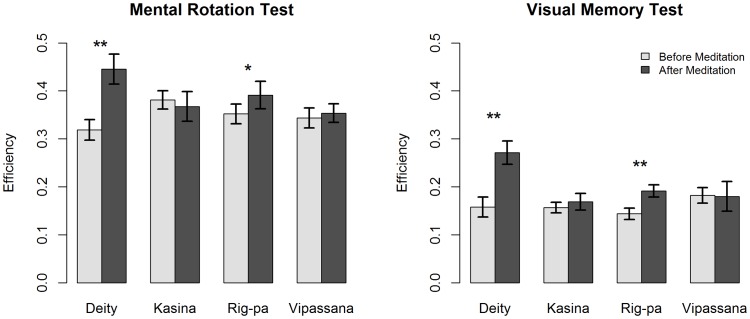
Efficiency scores before and after Meditation for the MRT (left panel) and VMT (right panel) tasks.

The MRT results were analyzed through a 2 (Time: pretest vs. post-test) X 4 (Condition: Kasina/Vipassana/Deity/Rig-pa) ANOVA, which indicated a significant main effect of Time, F(1,47) = 14.73, p<0.001. The effect of Condition was not significant, F<1. However, there was a significant interaction between Time and Condition, F(3,47) = 9.52, p<0.001. Follow-up ANOVAs revealed a Time effect for Deity, F(1,14) = 19.36, p<0.001, and Rig-pa, t(9) = 7.68, p<0.05, but not Kasina, F<1, and Vipassana, F<1 (see **Figure 7**).

#### VMT

Similarly to the MRT results, VMT were analyzed through a 2 (Time: pretest vs. post-test) X 4 (Condition: Kasina/Vipassana/Deity/Rig-pa) ANOVA, which showed a significant main effect of Time, F(1,45) = 20.73, p<0.001 (df = 45 as two Vipassana meditators did not perform the VMT). Again, the main effect of Condition was not significant, p>0.16, albeit a significant Time X Condition interaction was found, F(3,45) = 8.57, p<0.001. Follow-up ANOVAs revealed a significant efficiency increase for Deity, F(1,14) = 26.41, P<0.001, and Rig-pa, F(1,9) = 19.46, p<0.01, but not Kasina, F<1, or Vipassana, F<1, see **Figure 7**.

Overall, the results of Study 2 demonstrated a significant improvement in cognitive performance on two visual tasks, MRT and VMT, immediately after both Deity and Rig-pa Vajrayana styles of meditation, but not Theravada styles of meditation. Such dramatic improvements in cognitive performance in comparison with the baseline suggest enhanced phasic alertness takes place during Vajrayana meditation. The results are consistent with previous research on meditation [33] which demonstrated that, although the improvements are not long-lasting and return to baseline in about 20 min, certain types of meditation (e.g., Deity) can dramatically boost performance on visual tasks.

## Discussion

Whereas previous research mostly emphasized the power of Theravada styles of meditation to induce a relaxation response, even after short practices (e.g. 20 minutes) [2], [4]–[6], [80] as well as to promote tonic alertness [31], the findings of our research show that Vajrayana styles of meditation induce an arousal response during a comparable time interval, characterized by an increase in sympathetic activation, and promote enhanced phasic alertness. Specifically, Study 1 demonstrated that the meditators were more relaxed during Vipassana (Theravada) meditation practices than during rest, as manifested by increased HF for Vipassana meditation and decreased LF/HF and Alpha power. As for Kasina (Theravada), although we did not observe an HF increase, a decrease in LF/HF, decrease in Alpha power, and the overall similarity to the responses obtained for Vipassana also suggest a relaxation response. The opposite pattern of responses was observed for both Vajrayana practices. A decrease in HF power during Deity and Rig-pa meditations indicated that the meditators showed more arousal while engaging in Vajrayana meditation relative to rest. Additionally, only Vajrayana meditators demonstrated significantly enhanced performance on the MRT and VMT tasks following Vajrayana styles of meditation in Study 2. Such immediate dramatic improvements on cognitive task performance could only be attributed to enhanced phasic alertness due to arousal, which reflects rapid mobilization of resources to process stimuli and prepare the system for response [29], [71]. Moreover, our findings are consistent with those of a recent study that showed that the practice of g-Tummo Vajrayana meditation can lead to increases not only in peripheral but also in core body temperature [81], which is, to a large extent, mediated by increased sympathetic activation [82]. It is not likely that the enhanced performance we observed in our research is due to the fact that Vajrayana practice involves visualization, and thus results in more efficient metabolic processes necessary to successfully perform the visual imagery tasks administered to the practitioners. The practice of Rig-pa meditation, which is not a visualization type of meditation, produced improvements on the cognitive tasks, while the practice of Kasina meditation, which is a visualization type of meditation, did not lead to any improvements. Nevertheless, further studies are still needed in order to assess whether the performance enhancements which were observed in the current research are modality specific (effecting only visual performance). This should be examined in future studies through the administration of non-visual tests, such as verbal working memory or auditory attention tasks, before and after Vajrayana meditation.

It should be noted that although arousal and “fight-or-flight” or stress responses are related, since both result from the activation of the sympathetic system, they are not the same. Whereas arousal is “an energizing function” responsible for harnessing the body's resources for intense activity, fight-or-flight or the acute stress response (also called *hyperarousal*) is a physiological reaction that occurs in response to a perceived harmful event, attack, or threat to survival [83]. It is important to mention that while Vajrayana meditation activated the sympathetic system, it did not lead to a stress response, which according to recent studies [84]–[86] would be characterized by increases in Beta power. Our results showed not only that Vajrayana meditative practices led to significant decreases in Beta power, but also that these decreases were even larger than those that occurred to Theravada practitioners. Similarly, the relaxation response produced by Theravada or Vajrayana meditation cannot be reduced to a state of drowsiness or sleep, typically associated with increased Delta power [87], since meditators from both traditions exhibited Delta power decrease during their meditation. This suggests that both Theravada and Vajrayana styles of meditation are neither associated with drowsiness nor with stress, but rather that the meditators were in an alert and non-drowsy state. Furthermore, while it was shown in previous studies that Theravada styles of meditation can induce tonic alertness [31], the results of Study 2 demonstrated that Vajrayana styles of meditation induced phasic alertness as reflected by significant improvements on cognitive tasks immediately after Deity and Rig-pa practices.

An additional goal of our research was to re-examine the validity of the FA-OM classification vs. the classification based on arousal vs. relaxation responses that distinguishes between different meditative traditions. Our EEG results showed qualitatively different patterns of activation between Theravada and Vajrayana meditations, albeit highly similar activity between Kasina and Vipassana, and between Deity and Rig-pa. First, whereas both Theravada practices led to decreases in Alpha power, Vajrayana practices did not, consistent with recent studies that have established that relaxation is correlated with decreased Alpha power [77], [78]. Second, while we found a significant decrease in Beta power for both Theravada and Vajrayana meditations, it was significantly larger for Vajrayana. While increases in Beta power have been shown to occur during the active maintenance of current cognitive and sensorimotor states, decreases in Beta power have been attributed to the processing of upcoming stimuli [88], which is important for both Theravada and Vajrayana (e.g. noticing the content of one's mental activity). The more significant attenuation of Beta power in Vajrayana could be attributed to enhanced readiness to respond to stimuli during increased phasic alertness states, which occur during Vajrayana practices.

Third, whereas Vajrayana meditations showed a decrease in Gamma power relative to Rest, Vipassana meditation showed a Gamma power increase, consistent with a recent study that also demonstrated increased Gamma power during Vipassana [37]. Several other studies also reported increased Gamma power during meditations other than Vipassana. For instance, [12] found increased Gamma synchronization during loving-kindness meditation, which they interpreted to mean that neurons become more synchronized when meditators engage in this type of meditation (see also [89]). Although one must be careful when interpreting Gamma power in EEG, as some researchers believe it to be an artifact generated by miniature eye movements [90], there is nevertheless research that suggests that Gamma power might be linked to object representation and feature binding [91]–[94], as well as changes in awareness during meditation [37], and the current results might reflect the fact that Theravada and Vajrayana meditations produce states of awareness that are qualitatively different.

Fourth, while we observed only a marginally significant decrease in Alpha Coherence for both Theravada and Vajrayana meditations, we found a significant interaction between the attention and tradition classification of meditations. In particular, the increase in Alpha Coherence was larger for FA (Kasina) than OM (Vipassana) meditation for Theravada, but the opposite effect was observed for Vajrayana, where Alpha Coherence was larger for OM (Rig-pa) but not FA (Deity), inconsistently with the FA-OM classification. Considering that decrease in Alpha Coherence is associated with a more relaxed state [95], it is possible that Kasina requires more effort than Vipassana, while Rig-pa requires more effort than Deity.

Lastly, the difference in Beta Fronto-Central Coherence between Theravada and Vajrayana practices, manifested by an increase only during Vajrayana meditations, may be indicative of enhanced emotion evaluation and affective value choices [96]. This might be related to the emphasis on transformations of emotional states during Vajrayana versus the emphasis on nonattachment from emotions in Theravada practices [14], [97].

Thus, based on our findings, we suggest that the categorization of meditations as either “focused attention” or “open monitoring” cannot fully accommodate the range of behaviors and neural processes that are involved during meditation. Indeed, the utilization of focused or distributed attention is not clear cut for the majority of, if not all, meditation techniques, and both attentional systems are probably active at some level in most types of meditations. On the other hand, the distinction between relaxation and arousal is dichotomous: something either induces relaxation or arousal (or neither), but cannot induce both. Our data demonstrate reliable and consistent differences between meditative techniques across traditions, and that meditative practices can be appropriately classified on the basis of whether they produce relaxation or arousal. Importantly, neither relaxation nor arousal are simple by-products of the meditative practice, but play an essential role in the process of attaining insight, and the possibility that the insight obtained would differ depending on whether a relaxation or arousal approach is chosen ought to be investigated in future studies. Whereas the elicitation of an arousal or relaxation response cannot provide a complete description of any type of meditation, the results of the current research provide evidence that it is possible to reliably categorize different types of meditation in this manner.

The implications of our findings are important in several ways. First of all, the present research showed Vajrayana practices can lead to dramatic enhancement in cognitive performance, suggesting that Vajrayana practices can be useful in tasks where optimal performance is required. However, it is important to note that we did not assess long term (i.e. trait) effects of meditations in this research. While the improvements on cognitive tasks following Vajrayana meditation are immediate and dramatic, it is still to be investigated in further research whether they lead to long-term changes. At the same time, while such improvement were not observed after Theravada practices, there is evidence that Theravada meditations might lead to long-term improvement on attentional tasks [31], [98], [99].

Moreover, our results show that Theravada meditations produce relaxation responses, while Vajrayana types of meditation produce arousal, suggesting that Theravada meditations could be more appropriate for stress reduction than Vajrayana, while Vajrayana meditation could be more problematic in people with higher stress levels. However, it should be noted that our research was conducted on a sample of long-term practitioners. While previous research has shown that stress reduction can be achieved through Theravada types of meditation (i.e. Mindfulness Based Stress Reduction [100], [101]), even after just 4–5 weeks of training [102], [103], no studies have yet addressed how training in Vajrayana meditation affects stress levels. Thus, investigating how Vajrayana practitioners learn to respond to stressful situations as they gain additional meditation experience, especially practitioners with pre-existing stress or anxiety, is an important direction for future research. It is possible that during long-term practice, Vajrayana meditators develop unique strategies that can be utilized to cope with stressful situations, for instance by transforming their negative emotions into the positive emotional states of the visualized deity.

Limitations affecting the generalizability of our findings is the small sample size due to the difficulties in accessing experienced Vajrayana practitioners, as well as that meditators from different traditions did not have the same cultural background. Additionally, although our findings demonstrate that there are qualitative differences in the manner in which different types of meditation traditions influence the autonomic system, the degree to which Vajrayana and Theravada meditations activate the sympathetic and parasympathetic nervous systems, respectively, still requires further investigation. This could be done, for example, by assessing the level of epinephrine and nor-epinephrine that would be directly measured through blood samples taken before and after meditation. Furthermore, it would be an interesting research direction to explore whether the nature of the particular Deity that is meditated upon (e.g. peaceful, wrathful, etc.) would influence the level of arousal generated during Deity meditation. It is possible that wrathful Tantric Deities might generate higher levels of arousal in comparison to more peaceful Deities. In addition, since certain meditations utilize breathing specifically to alter the level of relaxation and arousal, it would be valuable for future studies to obtain concurrent respiration and EKG measures.

Despite the limitations, we were able for the first time to show that Vajrayana and Theravada styles of meditation are correlated with different neurophysiological substrates. More generally, the current findings undermine the prevalent view that all meditation practices bring about the same results, of enhancing cognitive performance and reducing stress levels. Indeed, it shows that the physiological and cognitive influences that meditations induce can vary greatly between traditions. Even though the benefits that can follow from different types of meditations of different traditions are often similarly described, leading to the widespread belief that they are in fact highly similar and that the choice to practice one meditation over another would not greatly influence the outcome of the practice, the current research shows not only that this is a misconception, but also that it has greatly hindered the progress of the scientific study of meditation. Our research shows that the large body of research on Theravada meditation is not generalizable to Vajrayana meditation, and thus Vajrayana practices should receive a greater emphasis in future research. Indeed, we show that the term “meditation” is in many ways too general, and have taken a step toward establishing a terminology that can appropriately distinguish the various practices from different traditions.

## Supporting Information

Table S1 **HRV Analysis.**
(DOCX)

Table S2 **Spectral Analysis.**
(DOCX)

Table S3 **Coherence Analysis.**
(DOCX)

Table S4 **MRT and VMT Analysis.**
(DOCX)

Supporting Information S1 **Brief Outline of Buddhist Traditions.**
(DOCX)
