# Supplementary material for: Arousal vs. Relaxation: A Comparison of the Neurophysiological and Cognitive Correlates of Vajrayana and Theravada Meditative Practices
Source: PLoS One. 2014 Jul 22;9(7):e102990. doi: 10.1371/journal.pone.0102990 (PMC4106862; doi:10.1371/journal.pone.0102990)
Supplement: Table S1 — HRV Analysis. (DOCX) [file pone.0102990.s001.docx]

**Table S1.** Heart Rate Variability Analysis

| **Meditation Tradition** | **Condition (FA/OM)** | **df** | **F** | **p** | **η_p_²** |
| --- | --- | --- | --- | --- | --- |
| **Theravada** | | | | | |
|  | HF | 2,18 | 3.20 | 0.06 | 0.26 |
|  | LF/HF | 2,18 | 3.67 | 0.046 | 0.29 |
| **Vajrayana** | | | | | |
|  | HF | 2,16 | 3.19 | 0.07 | 0.29 |
|  | LF/HF | 2,16 | 1.31 | 0.30 | 0.14 |
