# Supplementary material for: Arousal vs. Relaxation: A Comparison of the Neurophysiological and Cognitive Correlates of Vajrayana and Theravada Meditative Practices
Source: PLoS One. 2014 Jul 22;9(7):e102990. doi: 10.1371/journal.pone.0102990 (PMC4106862; doi:10.1371/journal.pone.0102990)
Supplement: Table S2 — Spectral Analysis. (DOCX) [file pone.0102990.s002.docx]

**Table S2.** Spectral Analysis

| **Meditation Tradition** | **Frequency** | **Condition (FA/OM)** | | | | **Location (L/R/C)** | | | | **Condition X Location** | | | |
| --- | --- | --- | --- | --- | --- | --- | --- | --- | --- | --- | --- | --- | --- |
|  |  | **df** | **F** | **p** | **η_p_²** | **df** | **F** | **p** | **η_p_²** | **df** | **F** | **p** | **η_p_²** |
| **Theravada** | | | | | | | | | | | | | |
|  | **Delta** | 2,18 | 8.37 | <0.01 | 0.48 | 2,18 | 7.19 | < 0.01 | 0.44 | 4,36 | 1.41 | 0.25 | 0.14 |
|  | **Theta** | 2,18 | 1.11 | 0.35 | 0.11 | 2,18 | 20.16 | <0.001 | 0.69 | 4,36 | 1.95 | 0.12 | 0.18 |
|  | **Alpha** | 2,18 | 6.84 | <0.01 | 0.43 | 2,18 | 13.49 | <0.001 | 0.60 | 4,36 | 2.90 | 0.04 | 0.24 |
|  | **Beta** | 2,18 | 3.68 | 0.046 | 0.29 | 2,18 | 0.28 | 0.76 | 0.03 | 4,36 | 3.77 | 0.01 | 0.30 |
|  | **Gamma** | 2,18 | 0.64 | 0.54 | 0.07 | 2,18 | 1.48 | 0.25 | 0.14 | 4,36 | 3.09 | 0.03 | 0.26 |
| **Vajrayana** | | | | | | | | | | | | | |
|  | **Delta** | 2,16 | 5.11 | 0.02 | 0.39 | 2,16 | 2.36 | 0.13 | 0.23 | 4,32 | 1.08 | 0.38 | 0.12 |
|  | **Theta** | 2,16 | 2.50 | 0.11 | 0.24 | 2,16 | 3.50 | 0.06 | 0.30 | 4,32 | 0.44 | 0.78 | 0.05 |
|  | **Alpha** | 2,16 | 0.22 | 0.80 | 0.03 | 2,16 | 0.20 | 0.82 | 0.02 | 4,32 | 0.09 | 0.99 | 0.01 |
|  | **Beta** | 2,16 | 8.42 | <0.01 | 0.51 | 2,16 | 3.43 | 0.06 | 0.30 | 4,32 | 1.01 | 0.41 | 0.11 |
|  | **Gamma** | 2,16 | 6.16 | <0.01 | 0.44 | 2,16 | 6.33 | <0.01 | 0.44 | 4,32 | 0.77 | 0.55 | 0.09 |
