# Supplementary material for: Arousal vs. Relaxation: A Comparison of the Neurophysiological and Cognitive Correlates of Vajrayana and Theravada Meditative Practices
Source: PLoS One. 2014 Jul 22;9(7):e102990. doi: 10.1371/journal.pone.0102990 (PMC4106862; doi:10.1371/journal.pone.0102990)
Supplement: Table S3 — Coherence Analysis. (DOCX) [file pone.0102990.s003.docx]

**Table S3.** Coherence Analysis

| **Meditation Tradition** | **Condition (FA/OM)** | **df** | **F** | **p** | **η_p_²** |
| --- | --- | --- | --- | --- | --- |
| **Theravada** | | | | | |
|  | Alpha Frontal | 2,18 | 2.74 | 0.09 | 0.23 |
|  | Alpha Fronto-Central | 2,18 | 2.04 | 0.16 | 0.19 |
|  | Alpha Posterior | 2,18 | 0.69 | 0.51 | 0.07 |
|  | Alpha Fronto-Posterior | 2,18 | 2.00 | 0.16 | 0.18 |
|  | Beta Frontal | 2,18 | 1.32 | 0.29 | 0.13 |
|  | Beta Fronto-Central | 2,18 | 0.23 | 0.80 | 0.03 |
|  | Beta Posterior | 2,18 | 0.40 | 0.67 | 0.04 |
|  | Beta Fronto-Posterior | 2,18 | 0.61 | 0.56 | 0.06 |
|  | Gamma Frontal | 2,18 | 0.20 | 0.82 | 0.02 |
|  | Gamma Fronto-Central | 2,18 | 0.37 | 0.70 | 0.04 |
|  | Gamma Posterior | 2,18 | 0.93 | 0.41 | 0.09 |
|  | Gamma Fronto-Posterior | 2,18 | 0.29 | 0.75 | 0.03 |
| **Vajrayana** | | | | | |
|  | Alpha Frontal | 2,16 | 3.20 | 0.07 | 0.29 |
|  | Alpha Fronto-Central | 2,16 | 1.25 | 0.31 | 0.14 |
|  | Alpha Posterior | 2,16 | 0.47 | 0.63 | 0.06 |
|  | Alpha Fronto-Posterior | 2,16 | 0.37 | 0.69 | 0.04 |
|  | Beta Frontal | 2,16 | 1.80 | 0.20 | 0.18 |
|  | Beta Fronto-Central | 2,16 | 3.34 | 0.06 | 0.30 |
|  | Beta Posterior | 2,16 | 0.77 | 0.48 | 0.09 |
|  | Beta Fronto-Posterior | 2,16 | 0.81 | 0.46 | 0.09 |
|  | Gamma Frontal | 2,16 | 2.62 | 0.10 | 0.25 |
|  | Gamma Fronto-Central | 2,16 | 1.10 | 0.36 | 0.12 |
|  | Gamma Posterior | 2,16 | 0.63 | 0.54 | 0.07 |
|  | Gamma Fronto-Posterior | 2,16 | 0.25 | 0.78 | 0.03 |
