# Supplementary material for: Arousal vs. Relaxation: A Comparison of the Neurophysiological and Cognitive Correlates of Vajrayana and Theravada Meditative Practices
Source: PLoS One. 2014 Jul 22;9(7):e102990. doi: 10.1371/journal.pone.0102990 (PMC4106862; doi:10.1371/journal.pone.0102990)
Supplement: Table S4 — MRT and VMT Analysis. (DOCX) [file pone.0102990.s004.docx]

**Table S4 – Study 2**

| **Task** | **Condition** | | | | **Time** | | | | **Condition X Time** | | | | |
| --- | --- | --- | --- | --- | --- | --- | --- | --- | --- | --- | --- | --- | --- |
|  | df | F | p | η_p_² | df | F | p | η_p_² | df | F | p | η_p_² |  |
| **Mental Rotation** | 3,47 | 0.46 | 0.71 | 0.03 | 1,47 | 14.73 | <0.0001 | 0.24 | 3,47 | 9.52 | <0.0001 | 0.38 |  |
| **Visual Memory** | 3,45 | 1.83 | 0.16 | 0.11 | 1,45 | 20.73 | <0.0001 | 0.32 | 3,45 | 8.57 | <0.0001 | 0.36 |  |
