# Supplementary material for: Arousal vs. Relaxation: A Comparison of the Neurophysiological and Cognitive Correlates of Vajrayana and Theravada Meditative Practices
Source: PLoS One. 2014 Jul 22;9(7):e102990. doi: 10.1371/journal.pone.0102990 (PMC4106862; doi:10.1371/journal.pone.0102990)
Supplement: Supporting Information S1 — Brief Outline of Buddhist Traditions. (DOCX) [file pone.0102990.s005.docx]

**Buddhist traditions**

Theravada, Mahayana, and Vajrayana (also called the “three vehicles”) are the main Buddhist traditions that exist today. Theravada, which has been the predominant religion of continental South Asia, draws its scriptural inspiration from the [*Tipitaka*](http://www.accesstoinsight.org/tipitaka/index.html), or *Pali canon* [[1](#_ENREF_1)], which scholars generally agree contains the earliest surviving record of the Buddha's teachings. Mahayana accepts the canonical texts of the Theravada tradition, but also introduces a vast corpus of philosophical and devotional texts, with the most distinctive feature of the great compassion, an inherent component of enlightenment, as manifested in bodhisattvas (enlightened beings). The Mahayana tradition is the largest major tradition of Buddhism (and includes Zen and Chinese Chan), and is prominent in North Asia. The third tradition is Vajrayana Buddhism, which is often referred to as Tantric Buddhism, and is a central tradition of Tibetan Buddhism. that adopted elements of Indian Tantric methods and Mahayana Buddhism [[2](#_ENREF_2), [3](#_ENREF_3), 5]. Although a number of practices in Tibetan Buddhism originated in Mahayana (e.g. training in compassion/Six Paramitas) and Theravada (e.g., renunciation, impermanence, elements of Shamatha and Vipassana), they are practiced and integrated in a Vajrayana context [[4](#_ENREF_4)].

**Relationship between Shamatha and Vipassana meditations**

While the Pali canon stresses that the practice of Shamatha is based on the idea of the development of “pacification”, “tranquility” or “serenity” of the mind, Vispassana requires the unity of serenity and insight, where insight either precedes or develops at the same time as serenity of the mind [[6: Mahavacchagotta Sutta (MN 73.18), Pindapataparisuddhi Sutta (MN 151.19)](#_ENREF_6)].

**References**

1. Snellgrove, D., *Indo-Tibetan Buddhism: Indian Buddhists and their Tibetan successors.* 2003, Boston, U.S.A.: Shambala.

2. Samuel, G., *The Origins of Yoga and Tantra: Indic Religions to the Thirteenth Century*. Vol. 2013. 2008, New Delhi, India: Cambridge University Press India Pvt. Ltd.

3. Harper, K.A. and R.L. Brown, eds. *The Roots of Tantra*. 2002, State University of New York Press: New York, U.S.A.

4. Panchen, N. and P.W. Gyalpo, *Perfect Conduct: Ascertaining the Three Vows*. 1999, Boston, U.S.A.: Wisdom Publications.

5. Thurman, R.A.F., *Tson-Kha-Pa's integration of Sotra and Tantra*, in *Soundings in Tibetan Civilization*, B.N. Aziz and M. Kapstein, Editors. 2008, Vajra Publications: Maryland, U.S.A. p. 372-382.

6. *Tipitaka: The Pali Canon*. 2005 [cited 2013 30 November 2013]; Legacy Edition:[Available from: <http://www.accesstoinsight.org/tipitaka/>.
